# A policy brief on improving the lifestyle of women with polycystic ovary syndrome

**DOI:** 10.22088/cjim.15.1.21

**Published:** 2024

**Authors:** Mouloud Agajani Delavar, Sedighe Esmaeilzadeh, Zynab Farhadi, Parvaneh Mirabi

**Affiliations:** 1 Infertility and Reproductive Health Research Center, Health Research Institute, Babol University of Medical Sciences, Babol, Iran; 2 Social Determinants of Health Research Center, Health Research Institute, Babol University of Medical Sciences, Babol, Iran

**Keywords:** Mentoring, Polycystic ovary syndrome, Lifestyle, Obesity, Family

## Abstract

Polycystic ovary syndrome (PCOS) in women is a significant public health issue. We searched the relevant databases using the sensitive keywords to receive the available evidence for successful lifestyle interventions among PCOS women. The systematic reviews related to PCOS were evaluated for an effective lifestyle intervention that was identified. The lifestyle interventions include three components: weight management, physical exercise, and behavioral coaching or combined interventions for developing exercise and modifying diet. Evidence shows that the impact of starting lifestyle intervention as the first-line management to improve obstetric and reproductive outcomes is high. There is evidence that proves health coaching can improve health behaviors and lifestyle. Thus, it is recommended to improve the lifestyle of women with PCOS.

## Executive Summary

polycystic ovary syndrome(PCOS) is the most common cause of anovulation in women of reproductive age (15-49 years), causing an ovulatory infertility (1, 2), which affect 5-18% of reproductive- age women (3). In addition, PCOS increases risk for chronic disease, psychological morbidity, and medication (3, 4). Obesity plays a central role in PCOS and leads to the development of PCOS and reproductive problems such as pregnancy loss and stillbirth, early gestational diabetes, and hypertension (5-8). Obesity is associated with increased visceral adipose tissue, which results in elevated androgen production, decreased sex hormone binding globulin, elevated hyperinsulinemia, and insulin resistance in females (9). It also might associate with disordered gonadotropin (GnRH) secretion which results in abnormal menstruation or amenorrhea (10). Some studies have shown that PCOS is associated with abnormal regulation of cholecystokinin and ghrelin hormones, that control appetite, and causes obesity (11-13).

In Iran, the problem of PCOS is among the priority issues of the healthcare system (14). Diagnosis of PCOS is according to Rotterdam diagnostic criteria by two features: polycystic ovarian morphology, clinical evidence of hyperandrogenism or biochemical, and oligo-ovulation/anovulation (Rotterdam ESHRE). The reason for this policy brief is to review recent available research evidence related to PCOS to provide a brief update on the improving lifestyle to PCOS women who are overweight/ obese.

## Context and importance of the problem

Today, lifestyle problem in developing countries has increased due to changed diet, lack of physical exercise, and adaption of a western lifestyle.

Therefore, the population of these countries is at higher risk for overweight/ obesity, and the importance of this problem is more common in women than men (15). 

Addressing effective lifestyle modification, as a patient priority, can provide better information for developing best- practice care in women with PCOS. In addition, the international evidence-based guideline for the assessment and management of PCOS also recommends lifestyle interventions as initial treatment. The goal of lifestyle interventions is to improve exercise or diet using implementing structured routines and structured approaches (16).

 The databases were searched using the sensitive keywords, to retrieve all review articles on PCOS and relevant policy documents. There is a wide range of review studies, and significant policies have conducted the management of PCOS and improve the lifestyle of women with PCOS (11, 17-37). However, despite that, still no agreement exists for a specific type of intervention. Therefore, arranging data on lifestyle health policy can have a great influence in reducing the disease burden on population health over a long period.

## Policy options

The importance of lifestyle interventions, including combining a healthy diet, increasing physical activity, and applying behavioral strategies, is well established as the first-line management for women with PCOS. Today healthcare workers strongly focus on counselling to control/ lose weight to improve adiposity, adiposity distribution, menstrual cyclicity, ovulation, hyperandrogenism, insulin resistance, diabetes mellitus, biochemical reproductive outcomes, and fertility (38). lifestyle intervention in overweight women with PCOS improves lipid profiles, metabolic status, maternal psychosocial status, quality of life, and cardiovascular disease (16, 39). Several practical ways to improve lifestyle have been planned. 

The first policy option in the healthy weight range, for overweight or obese women with PCOS is weight management based on PCOS international evidence-based guideline (EBG). Weight management is physiological processes and techniques to maintain a healthy weight, preventing weight gain, and achieving modest but meaningful weight loss. Effective weight management techniques include long-term lifestyle strategies that encourage the healthy eating and moderate physical activity every day (40). However, there is no substantial evidence to recommend the top dietary composition for women, with PCOS, but a balanced diet is recommended. In overweight women, it offers a slight weight loss of 5–10%. For achieving to this lose weight, the recommended deficit of 500- 750 kcal/day (30%) or around 1200–1500 kcal/day is the total intake according to individual energy requirements (16). 

The second policy option is physical exercise has been described that women with increased physical activity successfully maintain their weight loss. In addition, a structured exercise training program improves the frequency of menses, inflammatory markers, lipid profile, anthropometric measures, insulin sensitivity, and cardiopulmonary function in women with PCOS (41). It is generally recommended to have a minimum of 75 min exercise per week at a vigorous intensity 150 min of exercise per week at moderate intensity for general health benefits. For or more health benefits, prevention of weight gain, and modest weight loss, a 150-min of exercise per week at vigorous- intensity or 250 min of exercise per week at moderate intensity activities is endorsed (16).

 As well as, it is recommended to minimize the time in sedentary and strength exercises two alternating days per week (16, 42). 

The third policy option is behavioral management techniques for changing physical activity and weight management. Behavioral management techniques generally involve two phases; weight loss induction and weight maintenance. Generally, weight management and modifying lifestyle improve metabolic, anthropometric, and psychosocial outcomes, live birth, pregnancy, menstrual cycle, ovulation, and reproductive outcomes of women with PCOS (42-44). 

There is multiple behavioral strategies used for weight maintenance and weight loss, including self-monitoring, stimulus control, SMART goals (Specific, measurable, achievable, relevant, and time-bound), behavioral contracting/ reinforcement, nutrition education, meal planning, portion-controlled foods, modification of physical activity, social support, cognitive restructuring, and problem-solving (45).

 Despite the existence of these strategies, optimal behavioral strategies on how to achieve the recommended weight maintenance and weight loss are lacking. Strategies targeting improved motivation, social support, and psychological well-being are also essential. Some clear evidence suggests that health coaching is a holistic approach that involves a combination of behavioral interventions and psychological for managing behavior changes. It focuses an individual’s ability to change unhealthy behaviors by targeting psychological well-being, social support, and improved motivation (45-55).

 We used the key words including health behavior, health behavior management, health coaching, weight control, physical activity, nutrition, in PubMed, Google Scholar ,CINAHL, PsycINFO, Global Health, Psychology and Behavioral Sciences Collection to find all published assessing the effect of coaching on lifestyle outcomes in patients with various diseases. Details of eleven studies (50, 51, 56-64) for improving lifestyle outcomes in patients with various diseases are presented in table 1. The result of review trials of health coaching in patients with various diseases showed health coaching is a promising strategy for lifestyle improvements. 

**Table 1:** The effect of trials of coaching on lifestyle outcomes in patients with various diseases

**First author (year)** **Country**	**Population, mean age in year**	**Approach**	**Lifestyle Outcomes**
Saelens et al. (2002) (59) USA	Overweight and obese adolescents, Mean age coach: 14.2 y Mean age control: 19 y	Health coaching : 4-mo intervention, (11calls, weekly or biweekly), used computer program (calls Control: Typical care	Significantly increased mean BMI among control group. No differences between groups in weight-related behaviors and physical activity.
Vale et al. (2003) (57) Australia	Hospital patients with coronary heart disease Mean age Coach: 58.6y Mean age control: 58.3y	Coach: 6-mo intervention, 4 telephone coaching (20–30 min) (6-wk intervals) Control: no intervention	Significantly greater decreases in BMI, greater improvements in nutrient intake and greater waking among coach vs. control group.
Edelman et al.(2006) (61) USA	People with a chronic condition Mean age telephonic coaching : 52.2y Mean age control: 53.4y	Health coaching: 5-mo intervention, small group sessions and biweekly, individual telephonic coaching Control: Routine control	Significantly greater increased days of exercise, greater increase in physical activity among coach vs. control group.
Brodin et al. (2008) (50) Sweden	Patients with early rheumatoid arthritis Mean age coaching: 54.0y Mean age control: 56.0y	Health coaching: 1-y intervention, individual coaching and continuous telephone coaching Control: Routine control	Significantly greater improvements of muscle strength (physical activity) among coach vs. control group.
Paineau et al. (2008) (58) France	Parents and their children: aged 7–9 y Mean age coach: Children age: Group A = 7.7y Group B= 7.8y Group C= 7.6 y Parents: Group A= 40.4 y Group B=40.3 y Group C= 40.6 y	Family dietary coaching program by telephone calls: 8-mo intervention (Monthly) Group A: Low fat, high carbohydrate Group B: low fat, low sugar, high carbohydrate Group C: control groups	Nutritional target was achieved for fats in A and B, sugars decreased among group B compared with C, and total energy intake decreased in A and B among children and parents (B only). Significantly decrease in parent BMI and fat mass among group B vs. C. No significant difference in BMI and physical activity among children.
Grey et al. (2009) (62) USA	Students With BMI at 85th percentile and with family history of T2 diabetes Mean age coaching: 12.8 y Mean age control: 12.7 y	Coping skills training: (16-wk school-based intervention coping skills training, 9 -mo. of telephone health coaching	Significantly greater increased in BMI among coach vs. control group at the 12-mo follow-up. No significant differences between two groups in physical activity participation.
Wolever et al. (2010) (51) USA	Patients with type 2 diabetes Mean age coach: 53.1y Mean age control: 52.8y	Health coaching : 6-mo intervention, 14 telephone coaching (30-min) (1 initial call, 8 weekly calls, 4 biweekly calls, final call 1 mo later) Control: training motivational interviewing	Significantly greater increase in exercise and more medication adherence improved among coach vs. control group.
Bennett et al. (2010) (56) USA	patients with obesity and hypertension Mean age coaching: 54.4y Mean age control: 54.5y	Health coaching: 3-mo intervention (2 X 20 min) in-person coaching sessions and (2 x 20 min), telephone sessions Control: usual care	Significantly greater decreased in BMI in coach vs. control group. No significant differences between groups in waist circumference.
Hall (2019) (64) Indonesia	hypertension patients Age coach: 46-55 y Age control: 46-55y	Health coaching: 1-mo intervention, education and encouraged to make lifestyle for one month	Significantly greater increased in physical activity among coach vs. control group.
Shokri-Ghadikolaei et al. (2022 (63) Iran	postmenopausal and perimenopausal women Mean age coach: 50.9 y Mean age control: 49.8 y	Health coaching, five coaching sessions biweekly (30-45 mo.)	Significantly greater improved in quality of life and greater decreased in BMI, weight and waist circumference among coach vs. control group. No significant difference between two groups in physical activity of the participants.
Delavar et al. (2023) (60) Iran	Infertile overweight/obese women with PCOS Mean age coach: 28.3y Mean age control: 27.0y	Health coaching : 4-mo intervention, 6 calls (30-45min) Control group: Routine control	Significantly greater decreased in the waist circumference, greater increased in total physical activity, and greater improved in total quality of life among coach vs. control group.

BMI indicates body mass index, mo. indicates months, y indicates years

## Conclusion

The purpose of this policy brief was to define an effective lifestyle intervention for health professionals to guide women with PCOS who are overweight/ obese. Based on evidence, weight management is a significant concern for women with PCOS (65-69). Use lifestyle modification facilitators for weight management as a structured approach, such as having balanced meal support from health professionals, peers, friends and family seems warranted (22). 

Obstacles to changing lifestyle in women with PCOS include the following factors: 1) Lack of time and money 2) Motivational barriers such as fatigue or feeling unrewarded 3) Environmental barriers such as lack of access to safe places for exercise 4) Emotional barriers such as having depressing and defeating and relational thoughts 5) Obstacles such as an unsupportive partner 6) Prioritize children's food preferences (43).

 However, there is a lack of data on how to raise compliance and adherence of patients in lifestyle modification programs. Our study suggests that health coaching can be applied to the clinical management for lifestyle modification of women with PCOS. In addition, a sufficient number of health coaching researches are needed to upgrade the evidence of practical aspects of health coaching to improve fertility in women with PCOS. 
